# High-Dose Rifampicin Mediated Systemic Alterations of Cytokines, Chemokines, Growth Factors, Microbial Translocation Markers, and Acute-Phase Proteins in Pulmonary Tuberculosis

**DOI:** 10.3389/fphar.2022.896551

**Published:** 2022-07-15

**Authors:** Gokul Raj Kathamuthu, Perumal Kannabiran Bhavani, Manjula Singh, Jitendra Kumar Saini, Ashutosh Aggarwal, Mohammed Soheb S. Ansari, Rajiv Garg, Subash Babu

**Affiliations:** ^1^ National Institutes of Health-NIRT-International Center for Excellence in Research, Chennai, India; ^2^ National Institute for Research in Tuberculosis (NIRT), Chennai, India; ^3^ Division of Epidemiology & Communicable Diseases, Indian Council of Medical Research, New Delhi, India; ^4^ National Institute for Tuberculosis and Respiratory Diseases, New Delhi, India; ^5^ Post Graduate Institute of Medical Education and Research, Chandigarh, India; ^6^ Bhagwan Mahavir Medical Hospital & Research Centre, Hyderabad, India; ^7^ King George’s Medical University, Lucknow, India; ^8^ Laboratory of Parasitic Diseases, National Institute of Allergy and Infectious Diseases, National Institutes of Health, Bethesda, MD, United States

**Keywords:** pulmonary tuberculosis, high-dose rifampicin, cytokines, chemokines, growth factors, acute-phase proteins, microbial translocation markers

## Abstract

High-dose rifampicin (HDR) is now undergoing clinical trials to improve the efficacy of anti-tuberculosis treatment (ATT). However, the influence of HDR in the modulation of different cytokines, chemokines/growth factors, microbial translocation markers (MTMs), and acute-phase proteins (APPs) in pulmonary tuberculosis (PTB) is not well known. PTB individuals were separated into three different arms (R10, R25, and R35) based on their rifampicin dosage. We examined the circulating levels of Type 1, Type 2, pro-inflammatory/regulatory cytokines, chemokines/growth factors, MTMs, and APPs at baseline and after completion of the second month of ATT by ELISA. The baseline levels of cytokines, chemokines/growth factors, MTMs, and APPs did not (except IL-5, IL-6, IL-17A, MCP-1, MIP-1β, GCSF, SAA, ⍺2 MG, Hp) significantly differ between the study individuals. However, at the second month, the plasma levels of Type 1 (TNFα and IFNγ), Type 2 (IL-4, IL-5, and IL-13), pro-inflammatory/regulatory cytokines (IL-6, IL-17A, IL-10, and GMCSF), and APPs were significantly decreased in R35 regimen- compared to R25 and/or R10 regimen-treated PTB individuals. In contrast, the plasma levels of IL-2, IL-8, MCP-1, MIP-1β, GSF, and MTMs were significantly increased in the R35 regimen compared to R25 and/or R10 regimen-treated PTB individuals. Overall, our data reveal that HDR could potentially be beneficial for host immunity by altering different immune and inflammatory markers.

## Introduction

Tuberculosis (TB) is still considered a serious public health crisis coupled with high morbidity and mortality worldwide (World Health Organization, 2020). TB disease remains symptomless for a long time period, however, often establishes with active inflammation after the host immune system is compromised (Pai et al., 2016). Duration for TB treatment consists of 6 months and it contains a combination of four drugs (rifampicin, isoniazid, pyrazinamide, and ethambutol) including rifampicin during the full treatment course (World Health Organization, 2010). Previous mice model data revealed that high doses of rifampicin can accelerate remedy and hasten the conversion of sputum culture (Jayaram et al., 2003; Steingart et al., 2011; Rosenthal et al., 2012; de Steenwinkel et al., 2013; Hu et al., 2015). Also, other studies *in vitro*, as well as pharmacokinetic/pharmacodynamic studies, have shown that an elevated dose of rifampicin kills the bacteria more quickly and could help in the prevention of the occurrence of resistance (Jindani et al., 2003). However, so far, a 600 mg daily dosage of rifampicin is preferred as the standard dosage (van Ingen et al., 2011). Recently, different clinical trials have estimated the different doses (10 mg (mg)-35 mg/kg) of rifampicin against *Mycobacterium tuberculosis* (Mtb) disease and calculated the sputum conversion and frequency of regimen-related adverse events (Boeree et al., 2015; Boeree et al., 2017; Velásquez et al., 2018; Seijger et al., 2019; Nabisere et al., 2020). Although one concern is the excess metabolic processes that occur via the pregnane-x receptor upon high dose rifampicin induction (Niemi et al., 2003) and are often associated with adverse events mainly hepatotoxicity. A meta-analysis study displayed that hepatotoxicity occurred in 2.7% of patients receiving rifampicin-containing drugs for TB disease (Steele et al., 1991). However, no studies have examined the role of high dose rifampicin in altering the circulating levels of diverse cytokines, chemokines, growth factors, microbial translocation markers (MTMs), and acute-phase proteins (APPs) in PTB disease.

Cytokines can potentially mediate detrimental effects such as higher inflammation, uncontrolled tissue damage, and delayed tissue repair in chronic infections including TB (Torrado et al., 2011; Stek et al., 2018). Especially, Type 1, Type 17, and IL-1 family cytokines are crucial for the host protection; whereas Type 2, and anti-inflammatory cytokines either induce immune-related pathology or mediate susceptibility against PTB disease (Cooper et al., 2011). Similarly, plasma chemokines can function as markers of infection severity and bacteriological load in PTB (Kumar et al., 2021). Systemic immune activation is an important function of TB disease caused by Mtb in humans (Goletti et al., 1996; Hertoghe et al., 2000). Persistent activation of innate immune response is allied with greater systemic levels of microbial products like MTMs [soluble CD14 (sCD14), intestinal fatty acid-binding protein (I-FABP), lipopolysaccharide (LPS), and LPS-binding protein (LBP)] and APPs [C-reactive protein (CRP), serum amyloid protein (SAA), and α-2 macroglobulin (α-2M), and haptoglobin (Hp)] (Nixon and Landay, 2010). Similarly, the higher prevalence rate of acute-phase protein (APPs) during inflammation is an important auxiliary tool for predicting the prognosis and diagnosis of disease (Vermeire et al., 2005). To determine whether increased rifampicin dosage has any significant contribution to the modulation of the above-indicated markers, we measured various cytokines, chemokines, growth factors, MTMs, and APPs levels in three different arms of rifampicin treated PTB individuals. Our findings suggest that increased rifampicin dosage could potentially modulate the plasma levels of cytokines, chemokines, MTMs, and APPs in PTB individuals.

## Results

### Increased Rifampicin Dosage Is Significantly Associated With Reduced Systemic Levels of Type 1 (Except IL-2) and Type 2 Cytokines at the Second Month

**FIGURE 1:**
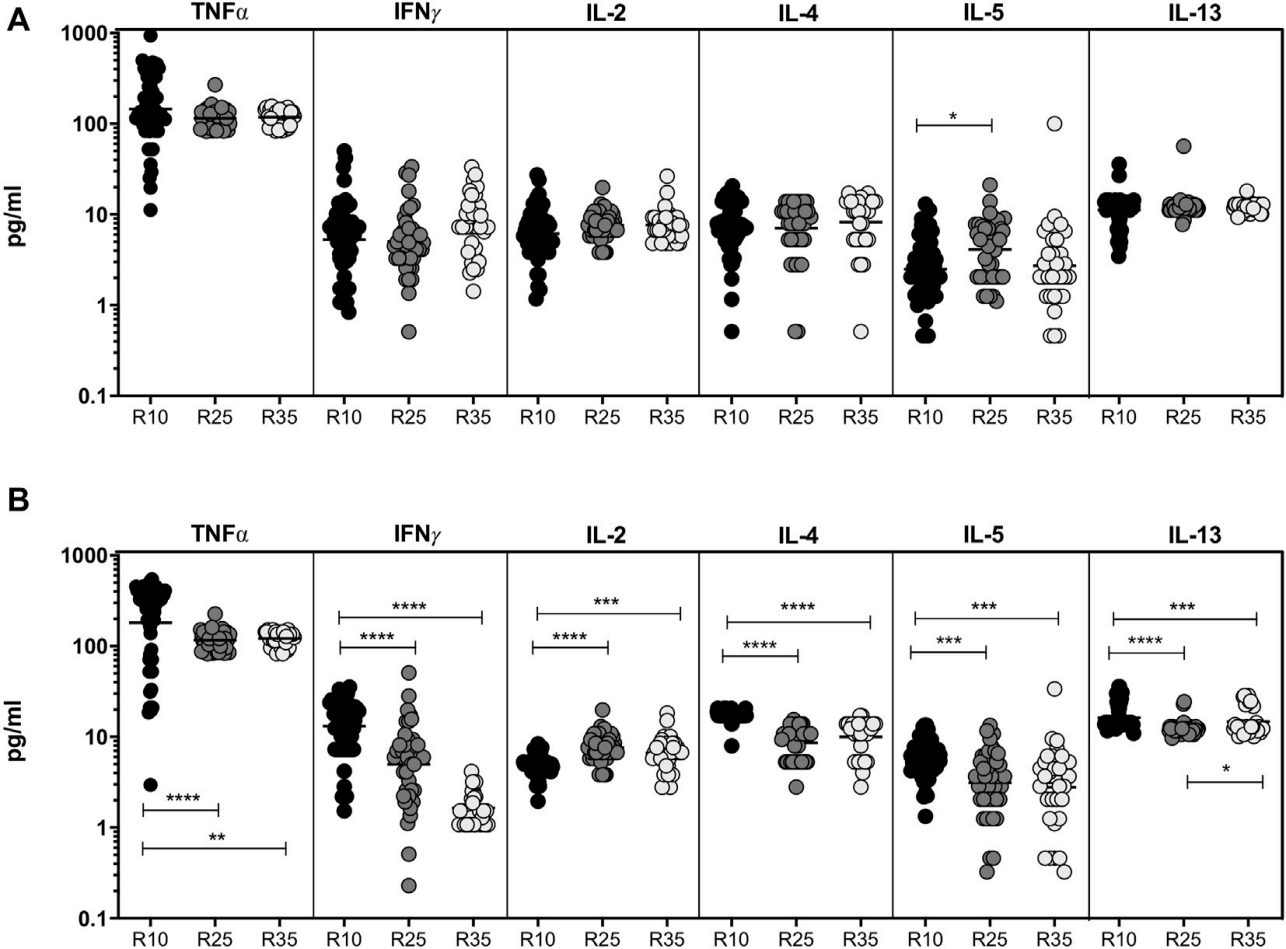
High dose rifampicin is associated with decreased plasma levels of Type 1 and Type 2 cytokines at the second month in PTB individuals. **(A)** Baseline analysis of Type 1 (IFNγ, TNFα, and IL-2) and Type 2 (IL-4, IL-5, and IL-13) cytokines between the three different rifampicin (R10 [n = 57], R25 [*n* = 42], R35 [n = 33]) treated arm of PTB individuals. **(B)** Second month analysis of Type 1 (IFNγ, TNFα, IL-2), and Type 2 (IL-4, IL-5, and IL-13) cytokines between the three different rifampicin (R10 [*n* = 57], R25 [*n* = 42], R35 [*n* = 33]) treated arm of PTB individuals. We used Kruskal–Wallis test with Dunn’s multiple comparisons to measure the *p* values and each individual were denoted using scatter plots.

To study the association of Type 1 (TNFα, IFNγ, and IL-2) and Type 2 (IL-4, IL-5, and IL-13) cytokines in PTB (different arms of rifampicin [R10, R25, R35] regimen) individuals, we have measured their plasma levels at baseline and the second month of ATT completion (Figure 1). As shown in Figure 1A, the baseline plasma levels of Type 1 and Type 2 cytokines were not significantly different (except IL-5, between R10, and R25) between the three different rifampicin regimen arms of PTB individuals. In contrast, the plasma levels of Type 1 (TNFα [geometric mean (GM) of 121.5 pg/ml in R35 arm compared to 116.3 and 180.9 pg/ml in R25 and R10 arm], IFNγ [GM of 1.65 pg/ml in R35 arm compared to 4.99 and 13.2 pg/ml in R25 and R10 arm]) and Type 2 (IL-4 [GM of 10.0 pg/ml in R35 arm compared to 8.5 and 17.1 pg/ml in R25 and R10 arm], IL-5 [GM of 2.79 pg/ml in R35 arm compared to 3.13 and 5.77 pg/ml in R25 and R10 arm], IL-13 [GM of 14.75 pg/ml in R35 arm compared to 12.42 and 16.30 pg/ml in R25 and R10 arm]) cytokines were significantly decreased and IL-2 was significantly elevated [GM of 6.63 pg/ml in R35 arm compared to 7.61 and 5.0 pg/ml in R25 and R10 arm] in R35 treated arm compared to R25 and R10 treated arms of PTB individuals (Figure 1B). Thus, the R35 treated arm of PTB individuals is associated with reduced plasma levels of Type 1 and Type 2 cytokines.

### Increased Rifampicin Dosage Significantly Alters the Pro-Inflammatory and Regulatory Cytokines at the Second Month

**FIGURE 2:**
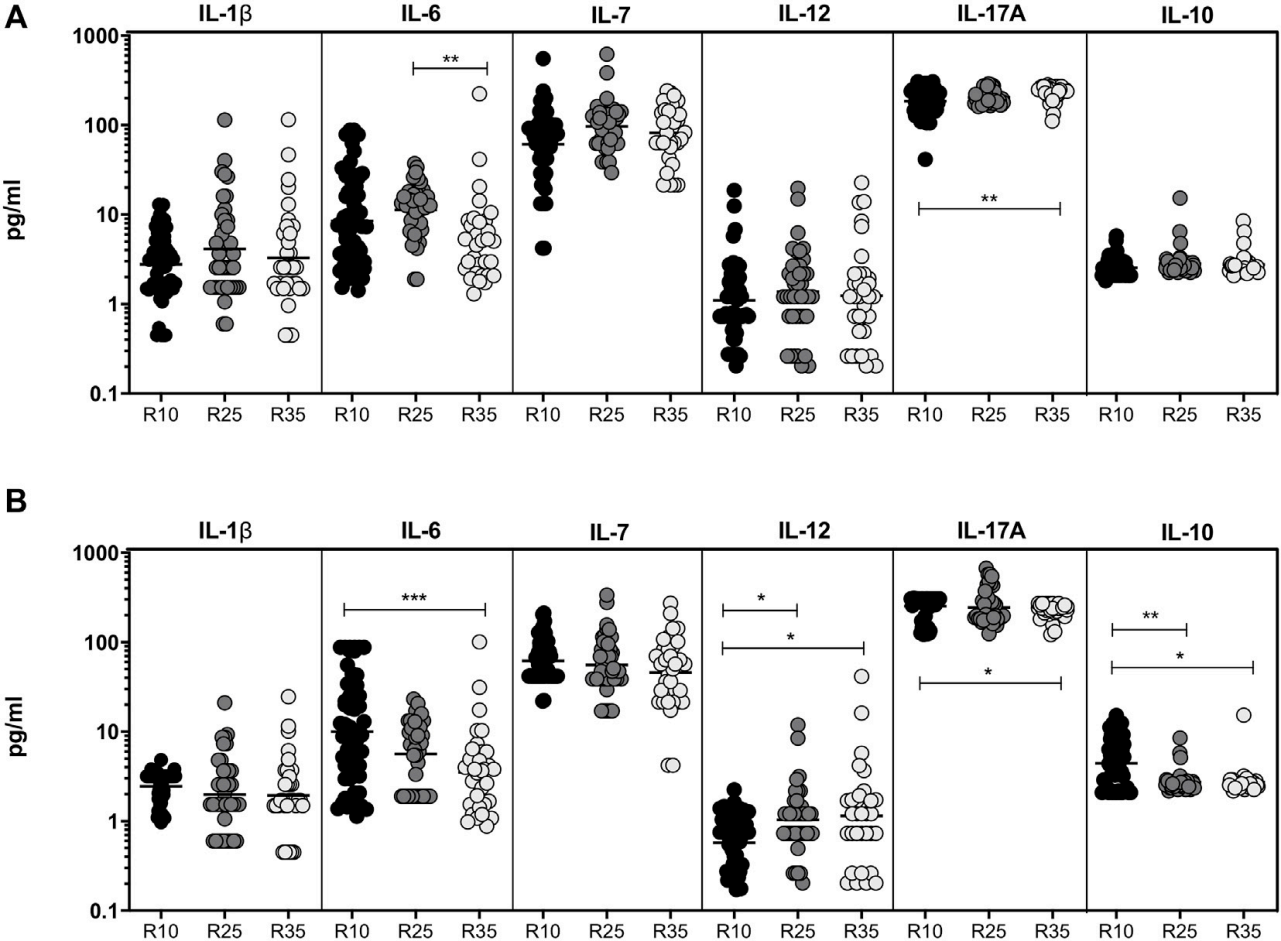
Altered pro-inflammatory and decreased regulatory cytokines are associated with High dose rifampicin treated PTB individuals at the second month **(A)** Baseline plasma levels of pro-inflammatory (IL-1β, IL-6, IL-7, IL-12, and IL-17A) and regulatory (IL-10) cytokines between the three different rifampicin (R10 [*n* = 57], R25 [*n* = 42], and R35 [*n* = 33]) treated arm of PTB individuals. **(B)** Second month plasma levels of pro-inflammatory (IL-1β, IL-6, IL-7, IL-12, and IL-17A) and regulatory (IL-10) cytokines between the three different rifampicin (R10 [*n* = 57], R25 [*n* = 42], and R35 [n = 33]) treated arm of PTB individuals. We used Kruskal–Wallis test with Dunn’s multiple comparisons to measure the *p* values and each individual were denoted using scatter plots.

To study the association of pro-inflammatory (IL-1β, IL-6, IL-7, IL-12, and IL-17A) and regulatory (IL-10) cytokines in PTB (different arms of rifampicin [R10, R25, and R35] regimen) individuals, we have measured their plasma levels at baseline and the second month of ATT completion (Figure 2). We show that at baseline the plasma levels of pro-inflammatory and regulatory cytokines are not significantly (except IL-6 and IL-17A) different between the three different rifampicin regimen arms of PTB individuals. In contrast, the plasma levels of pro-inflammatory and regulatory (IL-6 [GM of 5.239 pg/ml in R35 arm compared to 11.27 and 8.50 pg/ml in R25 and R10 arm], IL-17A [GM of 231.6 pg/ml in R35 arm compared to 204.5 and 184.4 pg/ml in R25 and R10 arm] and IL-10 [GM of 2.767 pg/ml in R35 arm compared to 2.837 and 2.547 pg/ml in R25 and R10 arm]) cytokines were significantly decreased and IL-12 was significantly elevated [GM of 1.24 pg/ml in R35 arm compared to 1.37 and 1.09 pg/ml in R25 and R10 arm] in R35 treated arm compared to R25 and/or R10 treated arms of PTB individuals. The other pro-inflammatory cytokines did not significantly differ between the study groups (Figures 2A,B). Thus, certain pro-inflammatory and regulatory cytokines were significantly altered in the R35 treated arm of PTB individuals.

### Increased Rifampicin Dosage Is Significantly Associated With Altered Systemic Levels of Chemokines and Growth Factors in the Second Month

**FIGURE 3:**
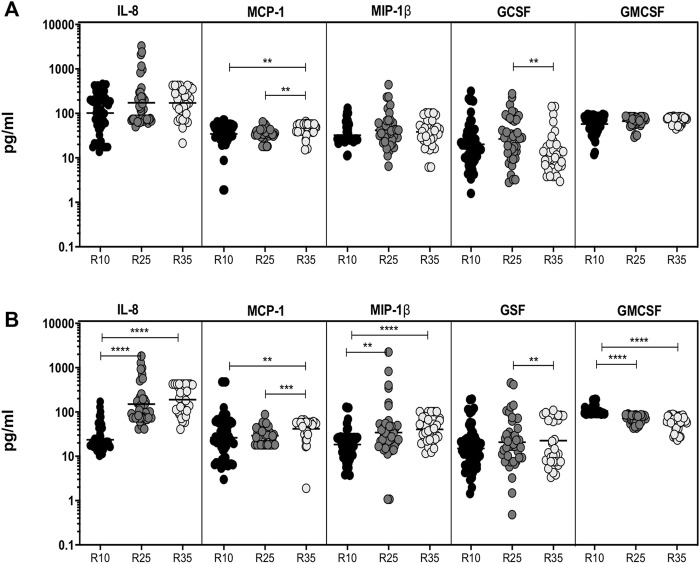
High dose rifampicin is associated with altered chemokines and growth factors at second month in PTB individuals. **(A)** Baseline plasma levels of chemokines (IL-8, MCP-1, MIP-1β) and growth (GCSF, GMCSF) factors between the three different rifampicin (R10 [*n* = 57], R25 [*n* = 42], R35 [*n* = 33]) treated arm of PTB individuals. **(B)** Second month plasma levels of chemokines (IL-8, MCP-1, MIP-1β) and growth (GCSF, GMCSF) factors between the three different rifampicin (R10 [*n* = 57], R25 [*n* = 42], R35 [*n* = 33]) treated arm of PTB individuals. We used Kruskal–Wallis test with Dunn’s multiple comparisons to measure the *p* values and each individual were denoted using scatter plots.

To study the association of chemokines (IL-8, MCP-1, and MIP-1β) and growth factors (GSF, GMCSF) in PTB (different arms of rifampicin [R10, R25, and R35] regimen) individuals, we have measured their plasma levels at baseline and the second month of ATT completion (Figure 3). As shown in Figure 3A, the baseline plasma levels of chemokines (except MCP-1) and growth factors (GCSF) are not significantly different between the three different rifampicin regimen arms of the PTB group. In contrast, IL-8 [GM of 172.7 pg/ml in R35 arm compared to 173.0 and 101.5 pg/ml in R25 and R10 arm], MCP-1 [GM of 43.58 pg/ml in R35 arm compared to 34.44 and 34.61 pg/ml in R25 and R10 arm] and MIP-1β [GM of 38.43 pg/ml in R35 arm compared to 41.68 and 32.35 pg/ml in R25 and R10 arm] plasma levels were significantly increased in R35 treated arm compared to R25 and R10 treated arms of PTB individuals. Similarly, GSF [GM of 12.79 pg/ml in R35 arm compared to 26.69 and 20.19 pg/ml in R25 and R10 arm] and GMCSF [GM of 71.42 pg/ml in R35 arm compared to 66.75 and 57.50 pg/ml in R25 and R10 arm] were significantly decreased in R35 treated arm compared to R25 and R10 treated arms of PTB individuals (Figure 3B). Thus, the R35 treated arm of PTB individuals is associated with altered plasma levels of chemokines and growth factors.

### Increased Rifampicin Dosage Is Significantly Associated With Elevated Systemic Levels of MTMs in the Second Month

**FIGURE 4:**
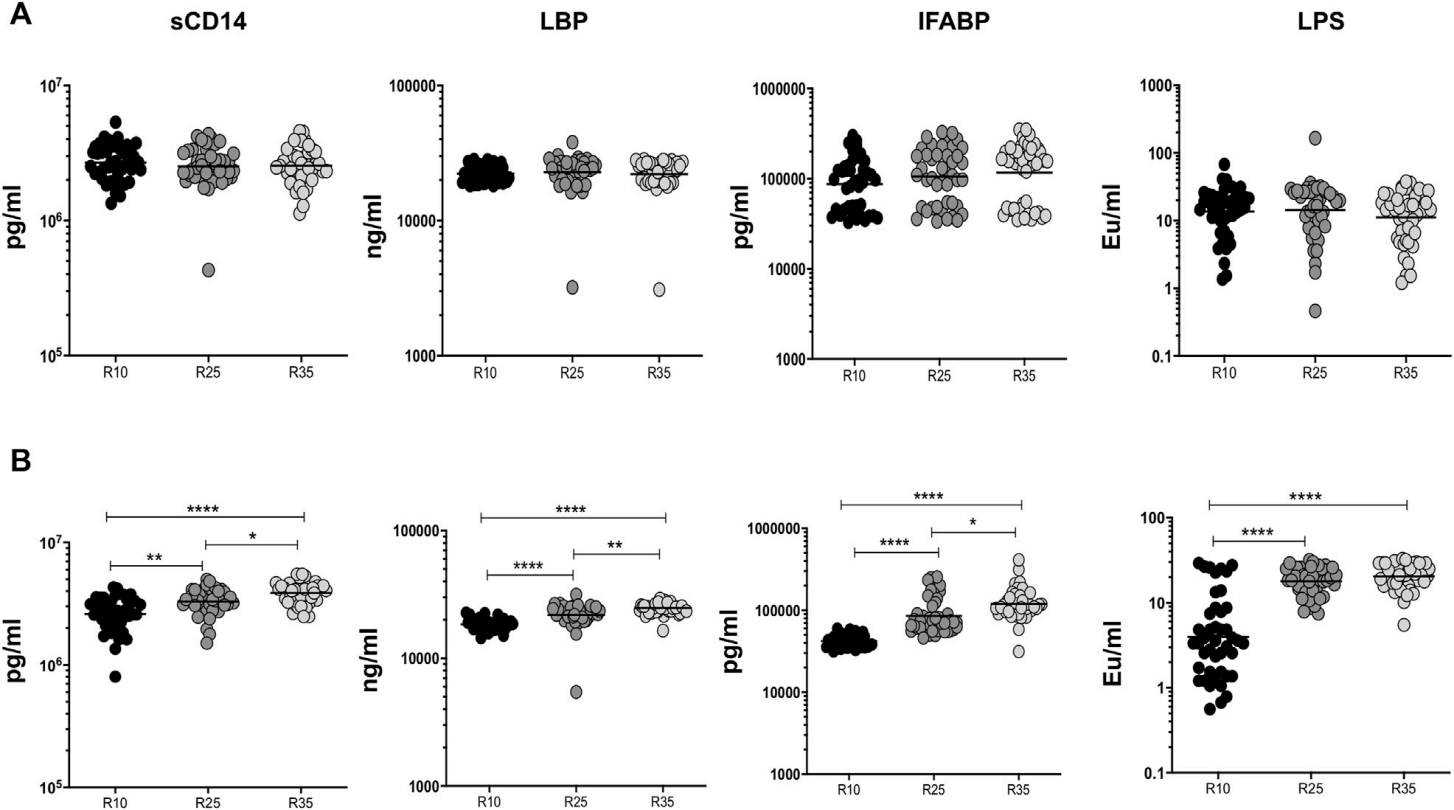
High dose rifampicin is associated with elevated MTMs at second month in PTB individuals. **(A)** Baseline plasma levels of MTMs (sCD14, LBP, IFABP, and LPS) between the three different rifampicin (R10 [n = 44], R25 [*n* = 44], R35 [*n* = 44]) treated arm of PTB individuals. **(B)** Second month plasma levels of MTMs (sCD14, LBP, IFABP, and LPS) between the three different rifampicin (R10 [*n* = 44], R25 [*n* = 44], R35 [*n* = 44]) treated arm of PTB individuals. We used Kruskal–Wallis test with Dunn’s multiple comparisons to measure the *p* values and each individual were denoted using scatter plots.

To examine the association of MTMs (sCD14, LBP, IFABP, and LPS) in PTB (different arms of rifampicin [R10, R25, and R35] regimen) individuals, we have measured their plasma levels at baseline and the second month of ATT completion (Figure 4). We show that at baseline the plasma levels of MTMs (sCD14, LBP, IFABP, and LPS) did not significantly alter in three different (R10, R25, and R35 regimen) arms of the rifampicin regimen given to PTB individuals (Figure 4A). However, in contrast, the plasma levels of MTMs [sCD14 (GM) of 3,867,576 pg/ml in R35 compared to 3,293,721 and 2,615,320 pg/ml in R25 and R10 regimen), LBP (GM of 24,784 ng/ml in R35 compared to 21,842 and 18,459 ng/ml in R25 and R10 regimen), IFABP (GM of 1,19,567 pg/ml in R35 compared to 85,621 and 42,196 pg/ml in R25 and R10 regimen) and LPS (GM of 11.16 enzyme units (Eu)/ml in R35 compared to 14.33 and 13.69 Eu/ml in R25 and R10 regimen)] were significantly elevated in (second month) R35 regimen arm compared to R10 and/or R25 regimen arm of PTB individuals (Figure 4B). Thus, the R35 treated arm of PTB individuals is associated with increased plasma levels of MTMs.

### Increased Rifampicin Dosage Is Significantly Associated With Diminished Systemic Levels of APP in the Second Month

**FIGURE 5:**
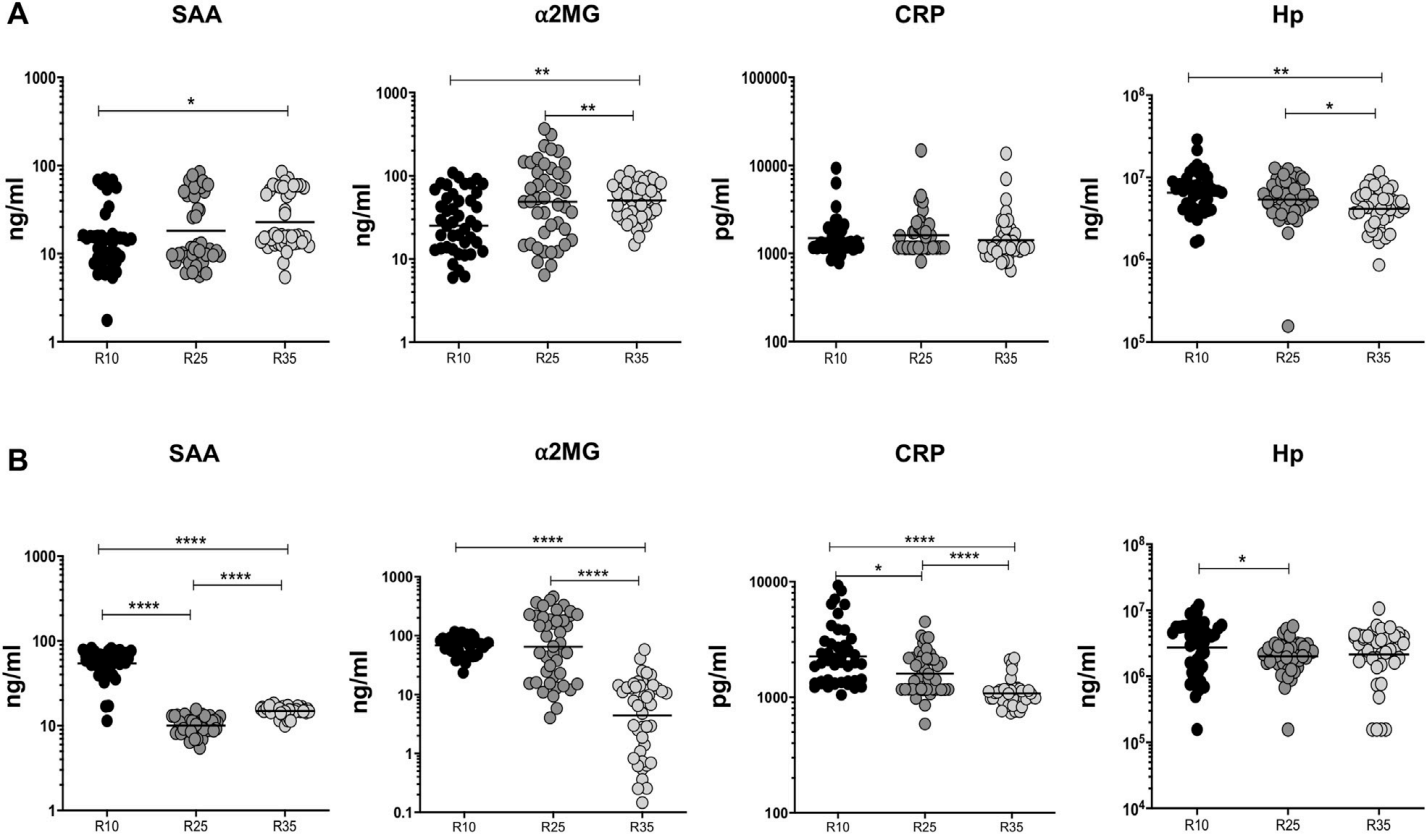
High dose rifampicin is associated with reduced APPs at the second month in PTB individuals. **(A)** Baseline plasma levels of APPs (SAA, α2MG, CRP, and Hp) between the three different rifampicin (R10 [*n* = 44], R25 [*n* = 44], R35 [*n* = 44]) treated arm of PTB individuals. **(B)** Second month plasma levels of APPs (SAA, α2MG, CRP, and Hp) between the three different rifampicin (R10 [*n* = 44], R25 [*n* = 44], R35 [*n* = 44]) treated arm of PTB individuals. We used Kruskal–Wallis test with Dunn’s multiple comparisons to measure the *p* values and each individual were denoted using scatter plots.

To examine the association of APPs (SAA, α2MG, CRP, and Hp) in PTB individuals, we have measured their plasma levels at baseline and the second month (different arms [R10, R25, and R35 regimen] of rifampicin) of ATT completion (Figure 5). We show that at baseline the plasma levels of CRP did not possess any significant difference between the different regimen treatment arms. However, in contrast, SAA (higher in R35 compared to R10), α2MG (higher in R35 compared to R10 and R25), and Hp (lower in R35 compared to R10 and R25) were significantly altered in three different (R10, R25, and R35 regimen) arms of rifampicin regimen given to PTB individuals (Figure 5A). Similarly, the plasma levels of SAA (geometric mean (GM) of 14.89 ng/ml in R35 compared to 10.07 and 54.26 ng/ml in R25 and R10 regimen), α2MG (GM of 4.402 ng/ml in R35 compared to 65.01 and 68.78 ng/ml in R25 and R10 regimen), CRP (GM of 1,078 pg/ml in R35 compared to 1,603 and 2,255 pg/ml in R25 and R10 regimen) and Hp (GM of 2,148,057 ng/ml in R35 compared to 2,006,633 and 2,743,895 ng/ml in R25 and R10 regimen) were significantly diminished in (second month) R35 regimen arm compared to R10 and/or R25 regimen arm of PTB individuals (Figure 5B). Thus, the R35 treated arm of PTB individuals is associated with reduced plasma levels of APPs.

## Discussion

Treating TB disease or patients with higher doses of rifampicin may be crucial since the drug effectively induces both bactericidal and sterilizing activity (Seijger et al., 2019). However, whether the high dose rifampicin can potentially be involved in the activation or suppression of different soluble immune markers is not known. Our results indicate that diverse cytokines, chemokines, growth factors, MTMs, and APPs were significantly altered during the high dose rifampicin treatment in the second month compared to their baseline levels.

Cytokines are primarily considered important mediators of resistance, with Type 1 cytokines (IFNγ, TNFα) being imperative for protection in TB disease (O'Garra et al., 2013; Cooper, 2009). But IFNγ alone is not adequate to confer protective immunity against TB; since their levels were not normally compromised (Casanova and Abel, 2002). In addition to IFNγ, TNFα plays a crucial role in the immune regulation against mycobacterial infection (Steele et al., 1991; O'Garra et al., 2013). Elevated Type 2 immunity has also been assumed to play an important function in susceptibility to TB infection (Deretic and Levine, 2009) and both IL-4 and IL-13 cytokines are shown to regulate Th1 mediated immune responses and to drive inappropriate alternate macrophage activation (Rook, 2007; Liu and Modlin, 2008; Deretic and Levine, 2009). Increased type 1 cytokines could possibly induce or extend the underlying immune-mediated pathology against TB infection (Cicchese et al., 2018; Stek et al., 2018). Our data show that Type 1 and Type 2 cytokine plasma levels were significantly reduced upon supplementation of high dose rifampicin indicating that the pro-inflammatory cytokine milieu, disease severity, and pathogenesis could potentially be attenuated upon treatment with high dose rifampicin. Both IFNγ and TNFα are associated majorly with inflammation and decreased levels might suggest that the inflammatory nature has been compromised upon treatment with a high dose of rifampicin. Therefore, on a clinical note, they might be utilized as a potential marker of altered inflammatory responses while monitoring anti-TB treatment.

Both, the IL-1 family and IL-12 are essential for driving Type 1 immune responses (Mayer-Barber and Sher, 2015). The occurrence of hereditary mutation in the IL-12/IFNγ pathway can increase the vulnerability to mycobacterial diseases (Casanova and Abel, 2002). High dose intravenous delivery of Mtb results in the expansion of pro-inflammatory (IL-6) cytokine, however, it is unessential in restricting mycobacterial growth upon lesser aerosol dose dispensed infection (Ladel et al., 1997; Saunders et al., 2000). Type 17 cytokines mainly induce resistance to extracellular bacteria and fungi which are involved in host immune responses to TB disease. IL-17A is necessary for the granuloma establishment in both BCG and TB disease (Khader et al., 2007) and increased production might also prime to elevated immunopathology linked with neutrophils invasion and commencement of tissue destruction (Cruz et al., 2010; Torrado et al., 2011). IL-10 plays a crucial role in inhibiting the macrophage and dendritic cell (DC) function and assists in the regulation and activation of immunity (Redford et al., 2011). They also play an important function during chronic and latent stages of PTB disease (Sasindran and Torrelles, 2011) and were higher during the infection and promote disease reactivation (Deveci et al., 2005). Our data suggest that both IL-17A, IL-6 and IL-10 were significantly reduced and IL-12 plasma levels were increased in individuals who are treated with high dose rifampicin. Hence, we suggest a high dose of rifampicin is associated with alterations in the cytokine (IL-17A, IL-6, IL-10, and IL-12) levels and could possibly,\ modulate the inflammatory mediated immune responses in TB, and might also act as a marker (IL-12, IL-6, and IL-10) of treatment outcome.

In addition to cytokines, chemokines are vital in cell migration to TB-affected organs and are essential for TB control and constructive granuloma formation. Chemokine dysregulation can modify the equilibrium from defense to inflammation and TB disease pathology (Slight and Khader, 2013; Monin and Khader, 2014). Our data show that IL-8, MCP-1, and MIP-1β levels are reduced and GMCSF levels are elevated upon a high dose of rifampicin exhibiting that increased rifampicin treatment alters the chemokine and growth factor milieu in TB as well.

Bacterial translocation occurs due to the passage of microbes and/or microbial products from the gut lumen of the gastrointestinal (GI) tract into the host circulation in the absence of bacteremia (Brenchley et al., 2006). They can directly stimulate systemic immune activation and contribute to inflammation. IFABP reflects a disruption in epithelial integrity associated with chronic intestinal infections. LPS mainly found in the cell wall of Mtb with the ability to stimulate inflammatory/phlogistic responses upon an interface with different types of cells, especially immune cells. In our study, the plasma levels of sCD14, LBP, IFABP, and LPS were significantly different and higher in R35 rifampicin treated arm compared to the other two arms. Indeed, higher MTMs could bind to diverse microbial products to activate monocytes, stimulate inflammatory/phlogistic responses, higher antigen clearance, and disrupt the integrity of epithelial cells upon infections. Thus, a high dose of rifampicin promotes microbial translocation and intestinal dysbiosis in TB. The higher levels of MTMs might be an important clinical factor to serve as biomarkers of favorable anti-TB treatment and risk signatures of disease severity at baseline.

Likewise, APPs chiefly originate from the liver and their systemic levels are thought to be the replication of pro-inflammatory cytokine reactions (Nathella et al., 2022). In addition, they might be used as a marker for offering diagnostic and prognostic evidence (Baumann and Gauldie, 1990; Johnson et al., 1999). Our data clearly describes that baseline levels of α2MG, SAA and Hp were significantly altered in three different (R35 compared to R25 and/or R10) arms of PTB individuals. However, the plasma levels of α2MG, SAA and CRP, and Hp (only between R10 versus R25 arm and no difference was found between R10/R25 compared to R35 arm, and the reason is not known) were significantly reduced in second month in high-dose rifampicin-treated arm compared to less dose-treated arm. Thus, a high dose of rifampicin is also associated with the modulation of systemic inflammation. Hence, we suggest APPs are clinically important predictors of treatment monitoring due to dampened inflammatory response observed after treatment in TB disease.

Overall, we show that the alterations that happen in the plasma levels of diverse cytokines, chemokines, growth factors, MTMs, and APPs are the characteristic feature of high-dose rifampicin given to the PTB individuals. However, one of the main limitations of the study is not showing the data either using the healthy volunteers treated with high-dose rifampicin or household contacts of study participants. The other study limiting factors or exclusion criteria is we did not take into account age, AST, and ALT levels for measuring the cytokines/chemokines/MTMs. Nevertheless, our data indicate that a higher rifampicin dosage might suppress the inflammation and potentially activate the essential immune response for the host protection against Mtb.

## Methods

### Ethics Statement

The National Institute for Research in Tuberculosis (NIRT) Institutional Review Board (IEC2017015) approved the study. We received written informed consent from all study individuals. The present study is part of a clinical trial (clinical trial number-CTRI/2017/12/010951) entitled “Phase IIb open-label randomized controlled clinical trial to evaluate the safety, tolerability, pharmacokinetics and anti-bacterial activity of high dose rifampicin versus conventional dose of rifampicin when given along with standard anti TB therapy for pulmonary tuberculosis” (HICON-R).

### Study Design

The demographic details of the study individuals (three different arms) are given in Tables 1, 2. A total of 132 pulmonary tuberculosis (PTB) serum samples were used in this study and they were defined as positive on both liquid and solid cultures using the Mycobacteria Growth Indicator Tube (MGIT) and Lowenstein–Jensen media. Those individuals were separated into three different (rifampicin (R) 10, R25, and R35) arms based on the rifampicin dosage given to the patients. R10 is a control arm where patients are treated daily with 10 mg of rifampicin, isoniazid, pyrazinamide, and ethambutol for 2 months followed by rifampicin (10 mg), isoniazid, and ethambutol daily for 4 months. Similarly, R25 and R35 groups are the experimental arms; where the patients were treated daily with a high dose of rifampicin (either 25 mg/kg/body weight [R25 arm] or 35 mg/kg/body weight [R35 arm]), isoniazid, pyrazinamide and ethambutol for 2 months (intensive phase) followed by rifampicin (10 mg), isoniazid and ethambutol for 4 months (Continuation Phase). Serum samples were also collected after the completion of the second month of anti-tuberculosis treatment (ATT). All the PTB individuals had no previous record of TB disease or were under ATT during the time of enrolment and had an abnormal chest x-ray. All the participants involved in this present study were not under any steroid treatment.

**TABLE 1:** Demographics of the study population for cytokines, chemokines, and growth factors.

Parameters studied	R10	R25	R35	*p* values
Number of subjects recruited (n)	57	42	33	—
Gender (Male/female)	32/12	31/11	24/9	NS^a^
Median age in years (Range)	29.6 (19–58)	35.6 (18–60)	40.6 (18–60)	NS^c^
Height (cm)	160.9 (141–175)	161.8 (142–184)	159.8 (139–176)	NS^c^
Weight (kg)	45.8 (30–63.6)	49.6 (32–65)	45.9 (31.1–64)	NS^c^
BMI	17.7 (13.4–28.2)	19 (12.2–23.9)	17.7 (12.5–26.0)	NS^c^
Smear grade (1+/2+/3+)	27/24/6	20/14/8	25/7/1	0.03^a^
Biochemistry and hematology				
AST or SGOT	27.4 (11–92)	23.9 (11–65)	24.1 (7.1–67)	NS^c^
ALT or SGPT	23.8 (4–99)	21.5 (4.2–85)	20.8 (7–111)	NS^c^
Serum alkaline phosphatase (SAP)	115 (73–195.9)	119 (45–354)	106.2 (43–258)	NS^c^
Hemoglobin	11.7 (7.6–16.6)	12.0 (8.1–16.9)	11.9 (8.9–15.9)	NS^c^
RBC	4.7 (2.84–5.9)	4.41 (3.29–6.02)	4.3 (3.25–5.58)	NS^c^
WBC	9.4 (5.3–15.3)	10.1 (5.0–18.72)	9.7 (4.1–13.49)	NS^c^
Platelets	397 (211–703)	361.8 (166–560)	391.2 (66–668)	NS^c^

**TABLE 2:** Demographics of the study population for APPs and MTMs.

Parameters studied	R10	R25	R35	*p* values
Number of subjects recruited (n)	44	44	44	—
Gender (Male/female)	32/12	31/13	31/13	NS^a^
Median age in years (Range)	40.7 (19–58)	35.8 (18–60)	38.9 (19–60)	NS^b^
Height (cm)	160.4 (141–180)	161.4 (139–185)	159.9 (140–178)	NS^b^
Weight (kg)	47.5 (30–63.6)	47.4 (36.4–65)	46.6 (30.1–64)	NS^b^
BMI	18.5 (13.4–28.2)	18.3 (12.2–26.0)	18.1 (12.2–26.0)	NS^b^
Smear grade (1+/2+/3+)	24/20/0	28/12/4	29/12/3	0.99^a^
Biochemistry and hematology				
AST or SGOT	27.4 (11–92)	23.9 (11–65)	24.1 (7.1–67)	NS^b^
ALT or SGPT	23.8 (4–99)	21.5 (4.2–85)	20.8 (7–111)	NS^b^
Serum alkaline phosphatase (SAP)	115 (73–195.9)	119 (45–354)	106.2 (43–258)	NS^b^
Hemoglobin	11.7 (7.6–16.6)	12.0 (8.1–16.9)	11.9 (8.9–15.9)	NS^b^
RBC	4.7 (2.84–5.9)	4.41 (3.29–6.02)	4.3 (3.25–5.58)	NS^b^
WBC	9.4 (5.3–15.3)	10.1 (5.0–18.72)	9.7 (4.1–13.49)	NS^b^
Platelets	397 (211–703)	361.8 (166–560)	391.2 (66–668)	NS^b^

a Chi-square test.

b Kruskal Wallis test; NS, non-significant.

### Study Inclusion and Exclusion Criteria

The patients were included in the study with no prior ATT history (or <15 days of ATT), with at least 2 sputum smears positive (in which 1 sputum should be RMP/INH sensitive by GeneXpert/line probe assay (LPA) Express). The patients were excluded from the study if their body weight is lesser than 30 kgs or greater than 65 kgs, infected with hepatic or renal disease or liver disease, had psychiatric illness, history of seizures or loss of consciousness, were seriously ill (Karnofsky scale <50), seropositive for HIV antibodies, HBS Ag or hepatitis C virus antibody, pregnancy or lactation and diabetics on insulin treatment. In addition, the patients were also excluded from the study if they have elevated alanine aminotransferase (ALT >2.5 times x ULN), total bilirubin (>1.2 times x ULN), serum creatinine (>1.2 mg/dl), blood Urea (>43 mg/dl), hemoglobin (<7.0 g/dl) or platelet count (<150,000/mm3), or WBC (<4,500 cells/μL) levels.

### Cytokines, Chemokines, and Growth Factors

The systemic levels of tumor necrosis factor (TNF)-α, interferon (IFN)-γ, Interleukin (IL)-2, IL-4, IL-5, IL-13, IL-1β, IL-6, IL-7, IL-12, IL-17A, IL-10, IL-8, MCP-1, MIP-1β, and granulocyte colony-stimulating factor (G-CSF) and granulocyte-macrophage colony-stimulating factor (GMCSF) were measured using Luminex assay according to the manufacturer’s protocol (Multiplex ELISA, Bio-Plex Pro Human Cytokine 17-plex Assay, BioRad, Hercules, CA, M5000031YV).

### Microbial Translocation Markers

Serum samples might contain endotoxin inhibiting compounds. Hence, endotoxin was inactivated by heating the samples for 5 min at 75°C before the ELISA was performed. The systemic levels of soluble CD14 (sCD14) (R&D Systems, Minneapolis, MN, United States), Lipopolysaccharide (LPS, limulus amebocyte lysate [LAL] assay), LPS- binding protein (LBP), and intestinal fatty acid-binding protein (IFABP) levels were measured using ELISA (Cell Sciences Hycult Biotech, Canton, MA, United States) according to the manufacturer’s protocol.

### Acute-Phase Proteins

The APPs measured in the present study are C-reactive protein (CRP), serum amyloid protein (SAA), and α-2 macroglobulin (α-2M) [DuoSet ELISA from R&D Systems]; whereas, haptoglobin (Hp) was measured using the BioSource ELISA kit. The experiment was performed according to the manufacturer’s instructions.

### Data Analysis

For calculating central tendency, we used geometric means (GM). To measure the statistically significant differences between the three groups, the Kruskal-Walli’s test with Dunn’s correction for multiple comparisons were used. All the statistical analyses and graphs were plotted using Graph pad Prism 9.2 (GraphPad Software Inc., San Diego, CA).

## Data Availability

The original contributions presented in the study are included in the article/Supplementary Material; further inquiries can be directed to the corresponding author.
